# A unique 15-bp InDel in the first intron of *BMPR1B* regulates its expression in Taihu pigs

**DOI:** 10.1186/s12864-022-08988-6

**Published:** 2022-12-03

**Authors:** Zhexi Liu, Ran Xu, Han Zhang, Depeng Wang, Ji Wang, Keliang Wu

**Affiliations:** 1 Department of Animal Genetics and Breeding, National Engineering Laboratory for Animal Breeding, College of Animal Science and Technology, China Agricultural University, Beijing, China; 2 Key Laboratory of Animal Genetics, Breeding and Reproduction of the Ministry of Agriculture and Rural Affairs, College of Animal Science and Technology, China Agricultural University, Beijing, China

**Keywords:** Taihu Pig, *BMPR1B*, Estrogen Response Element, Causal Variant, Alternate Splicing, Prolificacy

## Abstract

**Background:**

*BMPR1B* (Bone morphogenetic protein receptor type-1B) is a receptor in the bone morphogenetic protein (BMP) family and has been identified as a candidate gene for reproductive traits in pigs. Our previous study in Taihu pigs found a specific estrogen response element (ERE) in the first intron of the *BMPR1B* gene that is associated with the number born alive trait. However, little is known about the mechanism by which the ERE regulates the expression of *BMPR1B* in the endometrium.

**Results:**

Here, a 15-bp InDel (insertion/deletion) (AGCCAGAAAGGAGGA) was identified as a unique variation in Taihu pigs, and was shown to be responsible for the binding of the type I receptor of estrogen (ESR1) to the ERE using dual-luciferase assays. Four *BMPR1B* transcripts (T1, T2, T3, and T4) were identified by 5′ RACE in endometrial tissue. Expression of T3 and T4 in the endometrium of Meishan pigs was significantly higher than in Duroc pigs during pregnancy. Luciferase assays showed that three distinct *BMPR1B* promoters may drive expression of T1, T3, and T4. Interestingly, ERE-mediated enhancement of T4 promoter activity significantly increased expression of Transcript T4 in the endometrium of Taihu pigs (*P *< 0.05). In contrast, the ERE inhibited activity of the T3 promoter and decreased expression of the T3 transcript in the Duroc background (*P *< 0.05). In summary, we identified a 15-bp InDel in the Taihu ERE that can be used as a molecular marker for the number born alive trait, characterized the 5′ untranslated regions (UTRs) of *BMPR1B* transcripts in the endometrium, and determined how the transcripts are processed by alternative splicing events.

**Conclusions:**

Our results provide a foundation for understanding the transcriptional regulation of *BMPR1B* and its contributions to the unique breeding prolificacy characteristics of Taihu pigs.

**Supplementary Information:**

The online version contains supplementary material available at 10.1186/s12864-022-08988-6.

## Background

Evidence suggests that pigs in Asia were domesticated about 9000 years ago [1]. Chinese pig breeds are recognized for their high prolificacy, exceptional meat quality, resistance to disease, and adaptation to living in sties [2]. Taihu pigs, which are indigenous to the Taihu Lake region in China, are particularly prolific, having high ovulation rates coupled with low rates of embryonic mortality [3]. The Taihu group include six well-recognized breeds; Meishan (MS), Erhualian (EHL), Fengjing (FJ), Jiaxing Black (JXB), Mi(MI), and Shawutou (SWT), all of which originated from the same ancestor [4]. A better understanding the genetics of the high-quality traits shared by Taihu pigs and the underlying mechanisms responsible for large litters will advance the selective breeding for desired traits in existing commercial breeds.

To date, candidate genes associated with litter size in pigs include the type I receptor of estrogen (*ESR1*) and the follicle-stimulating hormone beta-subunit (*FSH-β*) [5, 6]. Some genetic polymorphisms in *ESR1* have a favorable effect on litter size, and *FSH-β* functions in the maturation of follicles [7]. Bone morphogenetic protein receptor 1B (*BMPR1B*) has been identified as a candidate gene responsible for reproductive traits. In our previous study [8], the Erhualian and Fengjing genomes were re-sequenced, revealing the unique estrogen response element (ERE) within the first intron of the *BMPR1B* gene in these pigs as well as in other breeds from the Taihu Lake region. The ERE may control pig prolificacy via cis-regulation of *BMPR1B* expression [8]. *BMPR1B* is the major receptor in the BMP protein family, the *BMPR1B* receptor and BMPs are closely associated with mammalian reproduction and influence ovarian follicular development, early embryonic development, differentiation in the female reproductive tract and osteoblast differentiation [9–11]. For example, *BMP4* regulates the proliferation and apoptosis of granule cells by binding either *BMPR1A* or *BMPR1B* [12], and *BMP5* positively affects the preimplantation of blastocysts and the development of bovine embryos [13]. *BMPR1B* is a high prolificacy gene that regulates litter size [8] and appears to be involved in the development of the endometrium. For example, *BMPR1B*^−/−^ mice have defective endometrial development, resulting in infertility [14]. In addition, certain miR125b binding site variations up-regulate *BMPR1B* expression, thereby reducing the proliferation of abnormal endometrial cells [15].

Major determinants for the high prolificacy of Taihu pigs are low fetal mortality and high uterine capacity [7], which are related to growth and development of the endometrium. However, the role of *BMPR1B* in the endometrium during pregnancy is still poorly understood. Splicing variants of the *BMPR1B* gene are distributed differently in various pig breeds and tissues. *BMPR1B* gene variants are associated with fertility traits in domestic animals. One FecB mutation *BMPR1B* c.746 A > G significantly affects the ovulation rate in Booroola-Merino Sheep [9], and a missense mutation *BMPR1B* c.746 A > G in transgenic pigs has been associated with prolificacy [16]. Evidence also suggests that the mutation *BMP7* c.1569A > G in the 3′-untranslated region (3′-*UTR*) is associated with the litter weight trait in Large White pigs [17].

Genetic variants are useful molecular markers that often influence gene expression and phenotype through various mechanisms [18]. A growing numbers of studies have identified single nucleotide polymorphism (SNPs) and copy number variations (CNVs) associated with reproductive traits in Meishan pigs through next-generation sequencing [19]. Genetic variants located within introns can also regulate the expression of target genes by influencing alternative splicing (AS). In the present study, we focused on *BMPR1B*, which is related to litter size in Taihu pigs. Earlier, we had identified a unique ERE within the first intron of the *BMPR1B* that efficiently binds to ESR1 and regulates the expression of the *BMPR1B* gene. We also determined that specific SNPs in the Taihu-ERE are significantly associated with the number born alive trait [8]. However, since there are four SNPs in this region, and the ERE also contains a 15-bp InDel, the site that is directly responsible for ESR1 binding remains to be identified. As reported here, we have found that the 15 bp InDel is unique in Taihu pigs by genotyping a wide variety of pig breeds using PCR amplification and polyacrylamide gel electrophoresis. We also explored the ERE region in more detail by measuring binding efficiency between ERE and ESR1 using dual-luciferase activity assays, and the structures of *BMPR1B* transcripts were also examined in endometrial tissue from pregnant pigs using 5’RACE. Finally, we investigated the ERE-mediated regulation of *BMPR1B* transcription using dual-luciferase assays.

## Results

### PCR amplification and sequencing of 15-bp InDel variants within *BMPR1B* in different pig breeds

**Fig. 1:**
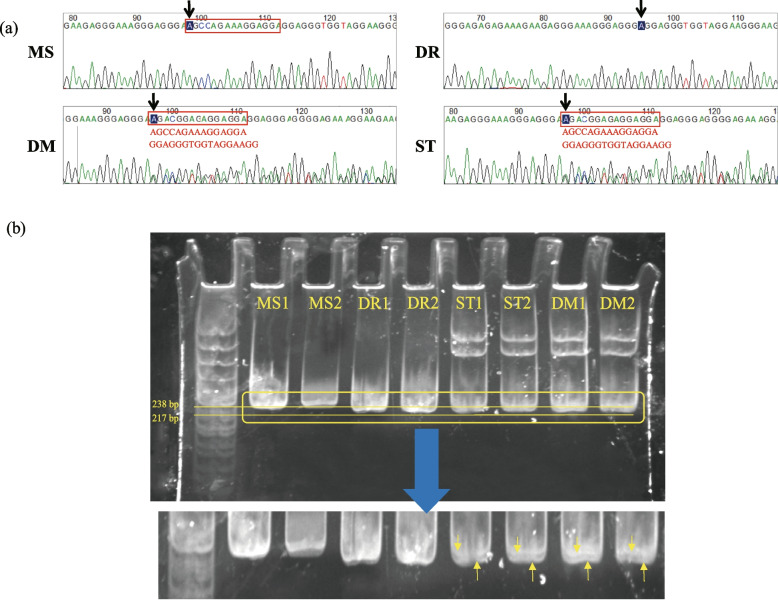
Identification of the 15-bp insertion sequence in the first intron of *BMPR1B* from four pig breeds. **a** Electropherograms generated by sequencing the *BMPR1B* InDel loci. Black arrowhead: variant sites; Red boxes indicate insertion sequences. Bases in red indicate that the InDel was present on only one chromosome. **b** Image of stained polyacrylamide gel. The image at the bottom is a magnified view of the area enclosed by the yellow box. The two thin horizontal yellow lines indicate the 238 bp marker (upper line) and the 217 bp marker (lower). In the magnified region, downward pointing arrows indicate bands containing the 15-bp InDel in hybrid pigs, while upward arrows indicate band that do not contain the InDel in hybrid pigs. “A” indicates presumptive heteroduplex. MS-Meishan, DR-Duroc, DM-Dumei, ST-Sutai

The Taihu unique estrogen response element (ERE) in the first intron of *BMPR1B* contains 4 SNPs and a 15-bp InDel (AGCCAGAAAGGAGGA). Locations of the SNPs and InDel are shown in Additional file 1: Table S1. To determine if the 15-bp InDel is unique to Taihu pigs, we genotyped 745 selected pigs representing various breeds (Table 1) by direct sequencing of the InDel. The results showed that the frequency of the homozygous insertion in the four Taihu breeds (Meishan, Erhualian, Jiaxing Black, and Shawutou) is extremely high, while the frequency of homozygous and heterozygous insertions in other indigenous and western pig breeds is low. PCR was also used to examine the InDel region in individuals from four breeds, including Meishan and Duroc pigs, and two Taihu x Duroc hybrids (using Sutai and Dumei pigs). The sequence electropherograms of the PCR products are shown in Fig. 1a. Because the litter sizes of Meishan and Duroc pigs are significantly different, the status of the *BMPR1B* InDel in these animals is of particular interest.

**Table 1:** Polymorphism of 15-bp InDel sequence in different pig breeds

Pig Breed (population size)	Insertion +/+	Insertion −/−	Insertion +/−	Insertion frequency	Allele frequency	
					Insertion+	Insertion−
**Meishan** (n=27)	27	0	0	1.00	1.00	0.00
Erhualian (n=13)	10	0	3	0.77	0.88	0.12
Jiaxing Black (n=27)	27	0	0	1.00	1.00	0.00
Shawutou (n=23)	22	1	0	0.96	0.96	0.04
Zang (n=28)	2	3	23	0.07	0.48	0.52
Jiangquhai (n=14)	2	4	8	0.14	0.43	0.57
Laiwu Black (n=49)	11	10	28	0.22	0.51	0.49
**Sutai **(n=56)	0	52	4	0.00	0.04	0.96
**Dumei** (n=47)	0	0	47	0.00	0.50	0.50
Saba (n=25)	0	1	24	0.00	0.48	0.52
Rongchang (n=36)	1	26	9	0.03	0.15	0.85
Wuzhishan (n=91)	0	71	20	0.00	0.11	0.89
Yimeng Black (n=29)	0	29	0	0.00	0.00	1.00
Guanling (n=15)	0	14	1	0.00	0.03	0.97
Xiang (n=78)	0	60	18	0.00	0.12	0.88
Zongdihua (n=36)	0	27	9	0.00	0.12	0.88
**Duroc** (n=37)	0	29	8	0.00	0.11	0.89
Yorkshire (n=54)	0	46	8	0.00	0.07	0.93
Landrace (n=60)	0	9	51	0.00	0.43	0.57

PAGE analysis of the PCR products from the four pig breeds revealed two bands around 242 bp in size, as expected for Sutai and Dumei pigs (Fig. 1b). Three genotypes (designated H1H1, H1H2, and H2H2) were associated with the 15-bp InDel. The single band at 242 bp in lanes 1 and 2 is the Taihu sequence (H2H2 genotype), the single band at 227 bp in lanes 3 and 4 is the Duroc sequence (H1H1 genotype), and the bands at 227 and 242 bp in lanes 5 through 8 are the heterozygous Sutai and Dumei sequences, respectively (H1H2 genotype). These results demonstrate the presence of a 15-bp InDel (AGCCAGAAAGGAGGA) in the first intron of *BMPR1B*. Note that heterozygotes exhibited 2 additional non-target bands in the PAGE analysis. The non-target bands were not characterized.

### Identification of variants affecting binding between the Taihu ERE and transcription factor ESR1

**Fig. 2:**
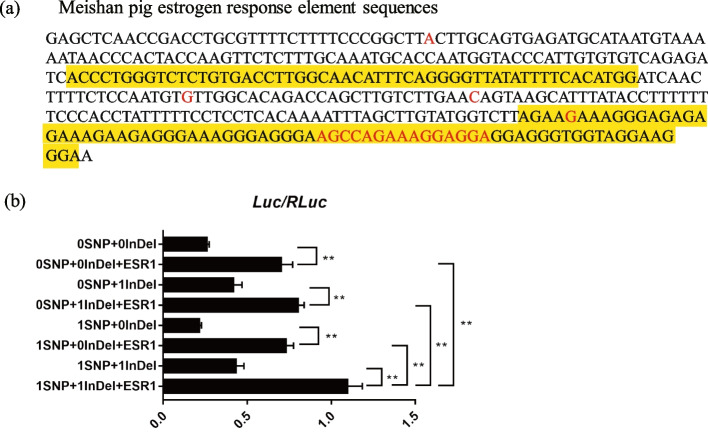
Identification of variants affecting differences in binding of ESR1 to Taihu-ERE. **a** Comparison of SNP and transcription factor ESR1 binding site sequences in Meishan and Duroc pigs. Red characters represent differences in sequence due to SNPs and the InDel. Yellow highlight indicates binding sites for transcription factor ESR1. **b** Relative luciferase activity generated by ESR1 binding to constructs containing SNPs and InDel sequences. Control constructs or constructs containing different combinations of variants are listed along the y-axis. The x-axis shows the luciferase activity normalized to reference luciferase activity. Error bars indicate means ± SEM. * indicates measurements that are significantly different (*P* < 0.05)

A previous study found that the Taihu ERE has significantly higher binding efficiency to transcription factor ESR1 (type I estrogen receptor) than Duroc ERE [8]. We predicted transcription factor binding sites in the Taihu ERE sequence using online tools at the AnimalTFDB3.0 website. One SNP (chr8: 134093159) and the InDel (AGCCAGAAAGGAGGA) within the Taihu ERE are both potential binding sites for ESR1 (Additional file 1: Table S2 and Fig. 2a). When luciferase assays were performed to compare the binding efficiencies of the SNP and InDel with ESR1, we found that the SNP + InDel generated significantly higher luciferase activity (Fig. 2b). Interestingly, in addition to ESR1, other transcription factors including SP1, STAT1, JUND, MYC, TP53, and NOTCH1 were predicted to bind the 15-bp InDel (Additional file 1: Table S2).

### Alternative splicing of *BMPR1B* transcripts

**Fig. 3:**
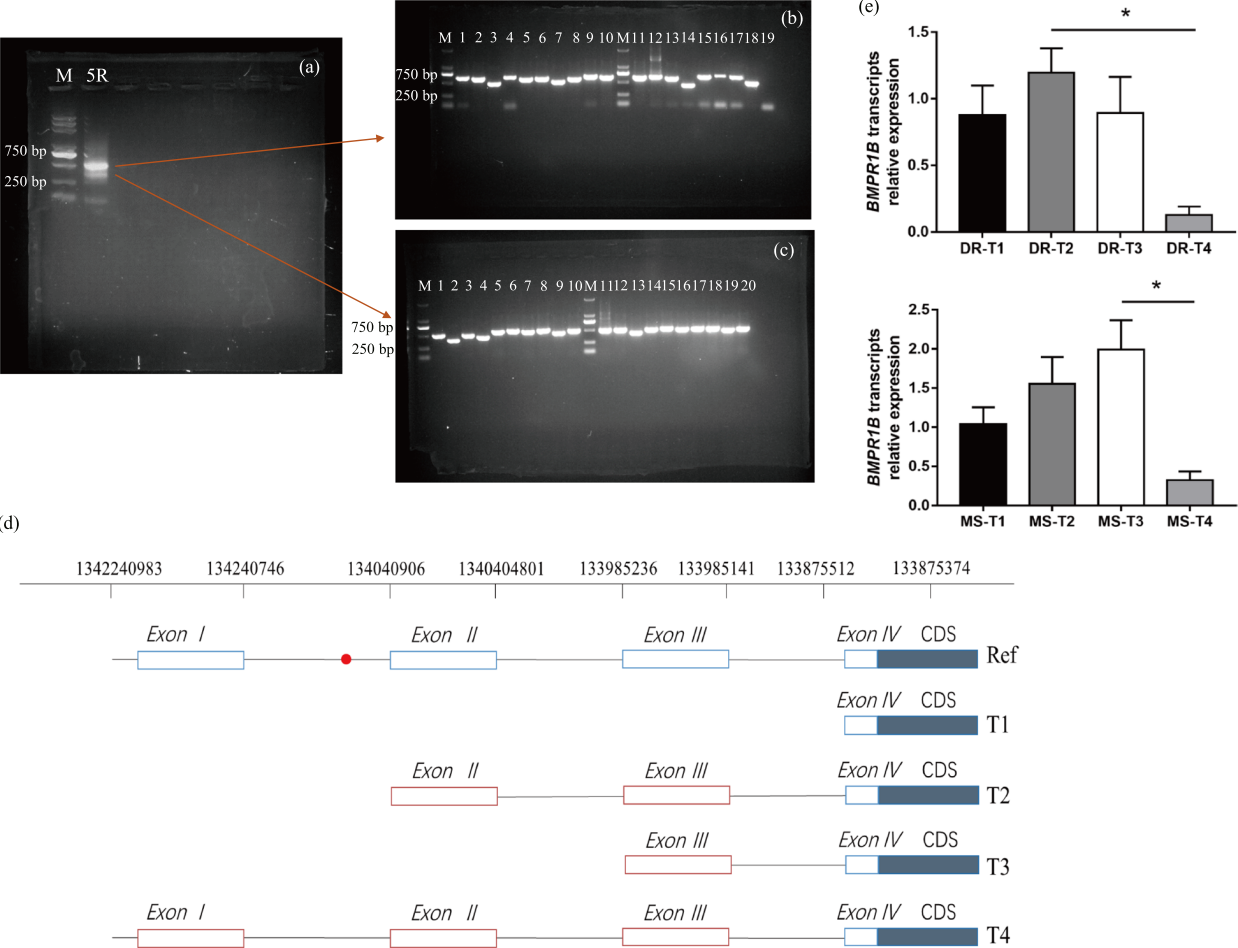
Alternatively spliced *BMPR1B* transcripts in endometrial tissue from pregnant sows. **a** Alternative splicing detected using 5′ RACE. **b** Fragments obtained by PCR amplification of cDNAs of approximately 500 bp in length. **c** Fragments obtained by PCR amplification of cDNAs of approximately 300 bp in length. **d** Diagram showing exon content in distinct *BMPR1B* gene transcripts. The alternative transcripts are numbered T1-T4 and the exons are designated. Blue boxes: conserved regions of the transcript; red boxes: alternatively spliced regions of the transcript. Ref (at the top) represents the full-length *BMPR1B* gene transcript. **e** The relative expression of different *BMPR1B* transcripts in Duroc (DR) and Meishan (MS) endometrium. * indicates measurements that are significantly different (*P* < 0.05)

The start codon of *BMPR1B* (Accession: NM_001039745.1 NCBI) in the *Sus scrofa* genome sequence is located in the fourth exon, suggesting that alternative splicing occurs during *BMPR1B* transcription. To investigate this possibility, we used 5′ RACE to synthesize and amplify the 5′ UTR of *BMPR1B* and found 300 bp and 500 bp cDNA fragments (Fig. 3a), consistent with two 5′-UTR variants or two transcriptions start sites in transcripts arising from *BMPR1B* in endometrial tissue. Additional analysis of the two cDNA fragments using PCR showed that there were indeed multiple alternative splicing events captured in the two cDNA fragments (Fig. 3b, c).

To characterize the alternative splicing in more detail, the two cDNA fragments were sequenced, and the results compared with the *BMPR1B* gene sequence. Four *BMPR1B* transcript variants were detected, designated T1-T4 (transcript sequences and locations are shown in Additional file 1: Table S3 and Fig. 3d). T1 contained exon 4, T2 contained exons 2, 3, and 4, T3 contained exons 3 and 4, and T4 contained exons 1, 2, 3, and 4. To confirm that the differences were due to alternative splicing rather than poor RNA quality, we performed RT-qPCR to quantify the transcript variants. In Duroc pigs, expression of T2 was significantly higher than T4, and in Meishan pigs, expression of T3 was significantly higher than T4 (Fig. 3e). The results show that the 4 *BMPR1B* transcripts are subject to differential expression in pregnant sows.

### Promoter prediction for the endometrial *BMPR1B* gene and detection of promoter activity

**Fig. 4:**
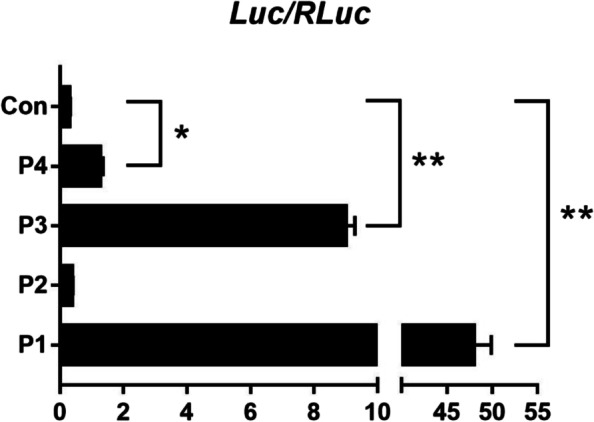
Promoter activity tests using dual-luciferase reporter system. Constructs containing different predicted promoters and controls are listed along the y-axis. The x-axis shows luciferase activity normalized to reference luciferase activity. Bars represent means ± SEM of the luciferase activity. Asterisks indicate values that are significantly different (*, *P* < 0.05; **, *P* < 0.01)

After confirming the presence of 4 *BMPR1B* transcript variants, we cloned and amplified four likely promoters (designated P1-P4). The promoter prediction scores for these regions are shown in Additional file 1: Table S4. Luciferase assays showed that the promoter activities (measured as luciferase activity) for pGL4.10-P1 (*P* < 0.01), pGL4.10-P3 (*P* < 0.01), and pGL4.10-P4 (*P* < 0.05) were significantly higher than that of the negative control pGL4.10-basic (Fig. 4), while the activity of the pGL4.10-P2 promoter was statistically indistinguishable from the negative control.

### The effect of Taihu-ERE on regulation of *BMPR1B* gene transcription and expression

**Fig. 5:**
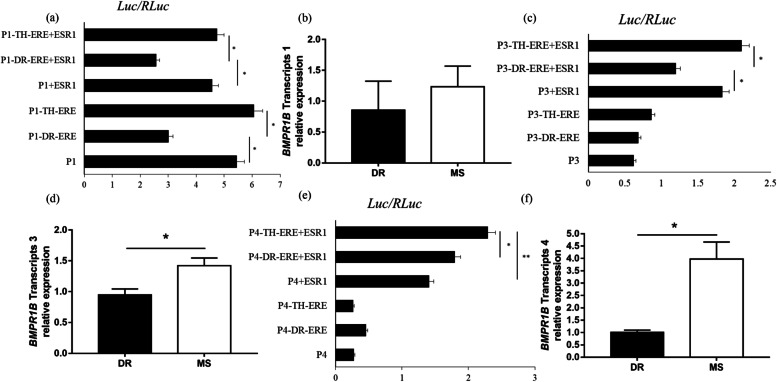
Regulation of *BMPR1B* promoter activities by the Taihu-specific ERE. **a** Effect of Taihu-specific ERE on promoter P1. **b** Expression of the T1 transcript. **c** Effect of Taihu-specific ERE on promoter P3. **d** Expression of the T3 transcript. **e** Effect of Taihu-specific ERE on promoter P4. **f** Expression of the T4 transcript. Data for a, c, and e were obtained using dual-luciferase reporter constructs, which are listed along the y-axis. The x-axis shows luciferase activity normalized to reference luciferase activity. mRNA expression data in b, d, and f were obtained by RT-qPCR (the y-axis shows relative expression). Bars indicate means ± SEM. Asterisks indicate values that are significantly different (*, *P* < 0.05; **, *P* < 0.01)

In a previous study, we found four Taihu-specific SNPs located within the conserved estrogen response element (ERE) in the first intron of *BMPR1B*. Taihu-ERE exhibited highly efficient binding to ESR1 and elevated the expression of *BMPR1B*. To determine how Taihu-ERE regulates *BMPR1B* transcription, we constructed four recombinant vectors containing Taihu/Duroc-ERE sequences and regions containing Promoters 1/3/4, and transformed them into Hela cells. The luciferase assay using Promoter 1 showed that activity of P1-DR-ERE was significantly lower than that of P1-TH-ERE (*P* < 0.05), and that there was no difference in the expression of *BMPR1B* transcript T1 in Duroc and Meishan pigs (Fig. 5a, b). The luciferase activity generated by P3-DR-ERE was significantly lower than that from P3-TH-ERE and P3-Basic (*P* < 0.05), and expression of transcript T3 in Meishan pigs was significantly higher than in Duroc pigs (*P* < 0.05) (Fig. 5c, d). Interestingly, the luciferase activity generated by P4-TH-ERE was significantly higher than by P4-DR-ERE (*P* < 0.05), and expression of transcript T4 in Meishan pigs was significantly higher than in Duroc pigs (*P* < 0.01) (Fig. 5e, f).

## Discussion

Livestock genetic resources serve both as primary materials for genetic improvement and important safeguards for sustainable breeding. They can also be used to explore the underlying molecular basis for the highly desirable traits found in some breeds. Reproductive capacity in pigs is a quantitative trait with low heritability that is controlled by multiple genes. Li identified a specific estrogen response element (ERE) in the first intron of *BMPR1B* in Chinese indigenous pig breeds from the Taihu Lake region; this element may promote binding with ESR1 to regulate *BMPR1B* gene expression [8]. Four SNPs and a 15-bp InDel are located in the ERE. However, the mechanism by which these structures contribute to the transcriptional regulation of *BMPR1B* by the Taihu-ERE required further investigation. In our investigation of the InDel polymorphism, we found that the frequency of homozygous insertion was over 75% in the Taihu pig population, but lower in other Chinese indigenous pigs and in breeds imported from western sources. Moreover, heterozygous insertions of the InDel were observed in Dumei and Sutai pigs, which are Duroc and Meishan hybrids. However, a higher number of individuals with heterozygous insertions were found in Dumei than in Sutai pigs. A possible explanation is that the Dumei pig is a developed line that contains 50% Duroc and 50% Meishan and is a first-generation descendant of the two breeds. In contrast, the Sutai pig has been bred more extensively [20] and may have been bred selectively to favor Duroc traits. Unexplained bands were also observed in Sutai and Dumei when PCR products were displayed using PAGE. These non-target bands may be heteroduplexes, consistent with previous studies [21, 22]. Interestingly, the InDel was also found in other Chinese indigenous pigs and western imports in heterozygous form, suggesting that a haplotype introgression event has occurred between Taihu pigs and other breeds. The Taihu pig is one of the oldest Chinese breeds, and the insertion sequence may have been introgressed into other breeds when they were hybridized with Meishan pigs to improve prolific performance [23]. Our data suggests that the 15-bp homozygous insertion (AGCCAGAAAGGAGGA) in the first intron of *BMPR1B* is unique to Taihu pigs.

The major classes of genetic variations include single-nucleotide polymorphisms (SNPs), insertions and deletions (InDels), and structural variation (SVs) [24]. In our study, the 15-bp InDel sequence as well as one SNP (chr8: 134093159) within *BMPR1B* were predicted as possible functional variants that could bind to transcription factor ESR1. As expected, the binding activity conferred by the SNP + InDel was significantly higher than that contributed by other variants, suggesting that the H2H2 genotype (InDel) exhibits significantly enhanced binding capacity with ESR1 in comparison with the H1H1 genotype. We conclude that the H2H2 genotype in the first intron of *BMPR1B* is the causal variant responsible for the binding of transcriptional factor ESR1. Our previous study identified ten SNPs (Taihu-specific haplotype) in the first intron of *BMPR1B* that were significantly associated with the number born alive trait [8], the InDel was closely linked with the SNPs (Taihu-specific haplotype) from our Sanger sequencing results. The 15-bp InDel was also located in this region and is also likely to affect the number born alive of pigs by enhancing the binding of ESR1. Thus, we inferred that the Taihu-specific 15-bp InDel is a convenient molecular marker to screen for the number born alive trait in pigs. Other studies have also demonstrated that InDels have utility as genetic markers to provide molecular targets for high-quality trait mining in animals [25]. An InDel sequence can regulate the transcriptional expression of downstream target genes by binding transcription factors or miRNA [26]. For example, the 13-bp InDel within the goat A-kinase anchoring protein 12 gene (*AKAP12*) might affect litter size by interfering with the binding of miR-181, thus influencing AKAP12 expression [27]. Li found that a 19-bp multiallelic InDel in the promoter region of the Cyclin-dependent Kinase Inhibitor 3 (*CDKN3*) gene is significantly associated with body weight and carcass traits in chickens [28]. Xu reported that a 10-bp InDel polymorphism in the promoter region of bovine Paired box 7 (*PAX7*) gene alters the binding of transcriptional factor ZNF219 and modulates promoter activity and expression of *PAX7* in Chinese cattle [29]. Finally, a 12-bp indel in the Cytokine inducible SH2-containing protein gene (*CISH*) potentially affects Landrace piglet susceptibility to diarrhea [30]. In our study, ESR1 was found to bind to the 15-bp InDel and regulate the expression of *BMPR1B*. Interestingly, binding sites for other transcriptional regulators, including JUND [31], SP 1[32], STAT 1[33], EZH 2[34], and TAF 1[35] were also predicted using computational methods, Moreover, potential binding sites for transcriptional factors involved in cell cycle regulation such as TP53 [36], MYC [37], and NOTCH1 [38] were identified, suggesting that the Taihu-specific InDel influences *BMPR1B* expression via multiple pathways to affect the growth and development of the endometrium.

Genomic variants within a non-coding region, such as a regulatory element, can result in a modified phenotype [39]. It is well established that alternative splicing (AS) can produce multiple transcript isoforms from the same gene, thereby increasing proteome diversity [40]. Previous reports raised the possibility that *BMPR1B* plays a key role in the development of the mammalian endometrium. However, the transcript repertoire of the *BMPR1B* gene in the endometrium during pregnancy in pigs was not explored. Because the 15-bp InDel variant is located in the first intron of *BMPR1B*, we hypothesized that the variant influences the transcription and expression of the gene by AS. Four *BMPR1B* gene transcripts were identified in the endometrium during pregnancy. We found that the T4 transcript included all exons, while T2 transcripts were missing exon I, T3 transcripts were missing exons I and II, and T1 transcripts were missing exons I, II, and III. At least three transcript variants of the 5′-UTR of the *BMPR1B* gene in Hu Sheep have been identified [41]. Abdurahman identified three transcriptional variants of *BMPR1B* in sheep ovarian follicles and showed that they were regulated by a feedback regulatory mechanism of the canonical BMP/Smad signaling pathway [41]. Together, these results demonstrate the important roles played by *BMPR1B* gene transcription variants. Since genetic variations can affect gene expression, they also can affect phenotypic characters. We examined expression of the mRNA splicing variants in endometrial tissue and showed that expression of *BMPR1B* Transcripts 3 and 4 was significantly higher in the endometrium of Meishan pigs, suggesting that these transcripts played an important role during pregnancy in this breed. Because these data are consistent with our results from the luciferase assay, we conclude that the Taihu-ERE enhances activity of the Transcript 4 promoter and significantly increases expression of this splicing variant in the endometrium. In contrast, the Duroc-ERE inhibits the activity of the Transcript 3 promoter, thereby decreasing expression of *BMPR1B* Transcript 3.

## Conclusion

Our study reveals that the 15-bp InDel within the first intron of *BMPR1B* is unique to Taihu pigs, and potentially affects the expression of *BMPR1B* during pregnancy by enhancing the binding of ESR1 and by regulating alternative splicing. We hypothesize that these mechanisms are responsible for increasing the number of pigs born alive in this breed. The InDel is therefore a valuable tool for MAS in pig breeding.

## Materials and methods

### Animals

All experimental animal protocols in this study were approved by the Animal Care and Use Committee of China Agricultural University (NO.AW010802202-1-1). A total of 745 pigs from 16 Chinese indigenous populations were used (including 4 from the Taihu Lake region), as well as 3 western domestic breeds.

### DNA and RNA extraction, PCR, and quantitative real-time PCR

DNA was extracted from ear tissue and blood using a Tiangen Genomic DNA Kit (DP204, Beijing, China). We genotyped a 15-bp InDel (insertion/deletion) by amplifying the appropriate genomic region using polymerase chain reactions. The products were then sequenced by Huada (Beijing, China). The position of the variants was determined by reference to the *Sus Scrofa* genome (version 10.2). Total RNA was isolated from the endometrium of Meishan and Duroc pigs 72 days after onset of pregnancy, following procedures described in a previous study [8, 42]. Primers are shown in Table 2. PCR conditions and cycling parameters were described previously [8], with exception of annealing at 56 °C. Relative gene expression was calculated according to the 2^-ΔΔCt^ method. Differential expression was analyzed using Student’s *t*-test with a significance threshold of *P* < 0.05. Before unpaired Student’s t-test, all samples were checked for normal distribution by SPSS (Windows, Version 25; IBM).

**Table 2:** Primers used in this study

Primer	Sequence (5′-3′)	Annealing temperature	Product size
*GAPDH*-F	GGGCATGAACCATGAGAAGT	56 °C	229 bp
*GAPDH*-R	AAGCAGGGATGATGTTCTGG	56 °C	
*BMPR1B*-T1-F	CCAGGTGATACTGATGTGC	56 °C	77 bp
*BMPR1B*-T1-R	CAGTGGTGGTGGCATTT	56 °C	
*BMPR1B*-T3-F	GCGGGTTTCAGAGTGGTCAAG	56 °C	94 bp
*BMPR1B*-T3-R	TGTATGGCAGGCTTGCTCCTTT	56 °C	
*BMPR1B*-T4-F	AACCAGTTGTCCCTGAGCTATGA	56 °C	132 bp
*BMPR1B*-T4-R	GGTGGCATTTACATCGCAAGA	56 °C	
*BMPR1B*-GSP	GATTACGCCAAGCTTGGACAGTGGTGGTGGCATT		
15-bp-InDel-F	CACAGACCAGCTTGTCTTGAA	56 °C	242 bp
15-bp-InDel-R	GGGCTCCTGTCTTTCTCTCT	56 °C	

### Polyacrylamide gel electrophoresis

PCR products were resolved on 12% polyacrylamide gels. Gels were prepared by mixing 5.9 mL ddH_2_O, 6 mL 30% polyacrylamide solution, 3 mL 5 × TBE buffer, 0.11 mL 10% ammonium persulfate, and 1 μL TEMED. After electrophoresis, bands were stained with Gel-Red (Tiangen, Beijing, China) and photographed for analysis.

### Rapid amplification of 5′ cDNA ends (5′-RACE)

The 5′-UTR sequence of porcine *BMPR1B* was amplified using a SMARTer RACE 5′/3′ cDNA amplification kit (Takara, Dalian, China), following the manufacturer’s instructions. Briefly, total RNA was extracted from endometrial tissue, reverse transcribed to convert the 5′ RNA ends to cDNA, and then 5′-RACE was performed using nested PCR with gene-specific primers and nested universal primers. Primer sequences are listed in Table 2. PCR products were separated on 1.5% agarose gels and recovered by extraction. Purified fragments were ligated into linearized pRACE vector (provided as a component of the SMARTer RACE 5′/3′ Kit). After sequencing and alignment to the genome (Huada, Beijing, China), the 5′ end of the porcine *BMPR1B* gene was identified.

### Promoter amplification and bioinformatic analysis

After identifying transcript variants from *BMPR1B*, we cloned and amplified promoters in the 2 kb upstream regions from transcripts T1-T4 (designated P1-P4, respectively). The corresponding DNA sequences were analyzed using online tools (https://wwwbimas.cit.nih.gov/molbio/proscan/) to locate potential promoters.

### Plasmid construction

To identify the variants responsible for the difference in binding efficiency between the estrogen response element (ERE) and the type I receptor of estrogen (ESR1), four pGL4.10 recombinant plasmids were generated: 1SNP-1InDel (Taihu-ERE), 1SNP-0InDel, 0SNP-1InDel, and 0SNP-0InDel (Duroc-ERE). Plasmid sequences were determined by Huada (Beijing, China).

To validate the activity of potential promoter regions, primers were used to amplify subregions within the potential promoter (primers are listed in Additional file 1: Table S5) using the sequenced plasmids described above as templates and the promoter-less luciferase reporter vector pGL4.10. Four pGL4.10 derivatives were generated, including pGL4.10-P1 (133,918,763-133,916,742), pGL4.10-P2 (134,150,195-134,148,947), pGL4.10-P3 (133,099,284-134,097,028), and pGL4.10-P4 (134,242,775-134,240,862).

To investigate the effect of Taihu-ERE on *BMPR1B* expression, two additional dual-luciferase reporter plasmids were constructed. Fragments containing the full-length estrogen response elements from the Taihu (TH) and Duroc (DR) breeds were amplified by PCR using primers listed in Additional file 1: Table S5. The TH-ERE fragment spanned coordinates 134,093,448-134,093,125, and the DR-ERE fragment spanned coordinates134,093,447-134,093,103. PCR was conducted as follows: 94 °C for 5 min, 40 cycles (95 °C for 30s, 58 °C for 30s, 72 °C for 30s),72 °C for 7 min. PCR products were purified using a Gel Extraction kit (OMEGA) and sub-cloned into the pGL4.10 promoter-less reporter vector to construct the reporter plasmids.

Gene-specific primers for pig ESR1 cDNA amplification were designed using NCBI reference sequence NM_214220.1. PCR products were cloned into the over-expression vector pCDH to construct pCDH-ESR1.

### Dual-luciferase assays

Transfection procedures were described in a previous study [8]. Briefly, 10^5^ Hela cells were cultured in a 24-well plate for 16-20 h. To perform the assay, pGL4.10 recombinant plasmids and pCDH-ESR1 were co-transfected into cells using Lipofectamine 3000 (Invitrogen, USA). The cells were also transfected with pRL-TK (a *Renilla* luciferase control reporter vector; Promega, USA). 4-6 h after transfection, 100 nM estradiol (Sigma, USA) was added to the medium. Luciferase activity was measured using the Dual-Glo Luciferase Assay System (Promega, USA).

To identify variants affecting binding between ERE and ESR1, Hela cells were transfected with 1000 ng pGL4.10 recombinant plasmids (1SNP-1InDel /1SNP-0InDel/ 0SNP-1InDel/0SNP-0InDel), 1000 ng pCDH-ESR1, and 100 ng pRL-TK.

To detect the activation of promoters driving different *BMPR1B* transcripts, 1000 ng pGL4.10-promoter recombined plasmids, 1000 ng pCDH-ESR1, and 100 ng pRL-TK were transfected into Hela cells.

To detect regulation by the Taihu-ERE on *BMPR1B* gene expression, 1000 ng pGL4.10-ERE and promoter plasmids (1000 ng pCDH-ESR1 and 100 ng pRL-TK) were transfected into Hela cells.

All experiments were repeated three times with three culture replicates each. Activities were calculated as mean ± standard error (SE).

### Transcription factor binding site prediction

Transcription factor binding sites were predicted using online tools at the AnimalTFDB3.0 website (AnimalTFDB3; hust.edu.cn). Binding sites were predicted for transcription factor ESR1 vs. MS-ERE and ESR1 vs. the Taihu unique 15-bp InDel.

### Statistical analysis

Results are presented as means ± SEM. Differences between means were analyzed using an unpaired Student’s t-test. Before unpaired Student’s t-test, all samples were checked for normal distribution by SPSS (Windows, Version 25; IBM). Comparisons involving more than 2 groups were analyzed by one-way ANOVA, Bonferroni correction for multiple comparisons were applied in one-way ANOVA (SPSS for Windows, Version 25; IBM). Differences were considered statistically significant at *P* < 0.05. Each experiment was repeated at least 3 times.

## Supplementary Information

**Additional file 1 : Table S1.** The location information for the SNPs and InDel

**Additional file 2 : Table S2.** The potential binding sites of transcriptional factors ESR1 in MS-ERE sequence

**Additional file 3 : Table S3.** The sequence information of *BMPR1B* transcripts

**Additional file 4 : Table S4.** The results of promoter predictions

**Additional file 5 : Table S5.** The information of primers used to construct plasmids in this study

## Data Availability

The variant data have been uploaded to the EVA database, the project ID is PRJEB56549 (https://www.ebi.ac.uk/ena/browser/view/PRJEB56549?show=analyses), and the analyses ID is ERZ14209126 (https://www.ebi.ac.uk/ena/browser/view/ERZ14209126?show=analyses). The data sets supporting the results of this article are included within the manuscript and its additional flies, the other data that support the study findings are available upon request to the authors (Email: liangkwu@cau.edu.cn).
