# Supplementary material for: A unique 15-bp InDel in the first intron of BMPR1B regulates its expression in Taihu pigs
Source: BMC Genomics. 2022 Dec 3;23:799. doi: 10.1186/s12864-022-08988-6 (PMC9719134; doi:10.1186/s12864-022-08988-6)
Supplement: Supplementary file 1 — Additional file 1 : Table S1. The location information for the SNPs and InDel [file 12864_2022_8988_MOESM1_ESM.docx]

Table S1 The location information for the SNPs and InDel

| Mutation location | Reference genome | Taihu genome |
| --- | --- | --- |
| Chr8 134,093,418 | G | A |
| Chr8 134,093,257 | A | G |
| Chr8 134,093,231 | T | C |
| Chr8 134,093,159 | A | G |
| Chr8 134,093,124-110 | - | AGCCAGAAAGGAGGA |
