# Supplementary material for: A unique 15-bp InDel in the first intron of BMPR1B regulates its expression in Taihu pigs
Source: BMC Genomics. 2022 Dec 3;23:799. doi: 10.1186/s12864-022-08988-6 (PMC9719134; doi:10.1186/s12864-022-08988-6)
Supplement: Supplementary file 3 — Additional file 3 : Table S3. The sequence information of BMPR1B transcripts [file 12864_2022_8988_MOESM3_ESM.docx]

Table S3 The sequence information of *BMPR1B* transcripts

| *BMPR1B* Transcripts | Sequence information |
| --- | --- |
| *BMPR1B* Transcripts 1 | GCAAACTTCCTTGATAACATGCTTTTGCGAAGTTCAGGAAAATTAAATGTGGGCACCAAGAAAGAGGATGGTGAGAGTACAGCCCCCACCCCTCGTCCAAAGATCTTGCGATGTAAATGCCACCACCACTGTCCAGAA |
|  |  |
| *BMPR1B* Transcripts 2 | GTTTCAGGGTGGCCCAGAGAGTGAACGCCTCTGAAGTGGATGTGCAGTGCGTAAATCATCCAGAAGGTCGTATGCTTCGGTTAGCAGCAGCCTGTTTATCTGGTTCCAACTTCTGCTGAATCACAACCAGTTGTCCCTGAGCTATGACAAGAGAGGAAACAAAAAGTTAAAGGAGCAAGCCTGCCATACACCAGAAGCAAATTTCCTTGATAACATGCTTTTGCGAAGTTCAGGAAAATTAAATGTGGGCACCAAGAAAGAGGATGGTGAGAGTACAGCCCCCACCCCTCGTCCAAAGATCTTGCGATGTAAATGCCACCACCACTGTCCAGAA |
| *BMPR1B* Transcripts 3 | GGTTCCAACTTCTGCTGAATCACAACCAGTTGTCCCTGAGCTATGACAAGAGAGGCAACAAAAAGTTAAAGGAGCAAGCCTGCCATACACCAGAAGCAAACTTCCTTGATAACATGCTTTTGCGAAGTTCAGGAAAATTAAATGTGGGCACCAAGAAAGAGGATGGTGAGAGTACAGCCCCCACCCCTCGTCCAAAGATCTTGCGATGTAAATGCCACCACCACTGTCCAGAA |
| *BMPR1B* Transcripts 4 | GGCGCGGCCGGGCGCGGAGTGGGCGCGGGGCCTGCGGGGCGCCAGGACGGGCCGGGGACCCGGGACCGCGGGGCGGCGGCGTGGAGGACGCCAGTGCCGGGGCGCAGCGGCGGGTTTCAGAGTGGTCAAGAGAGTGAACGCCTCTGAAGTGGATGTGCAGTGCATAAATCATCCAGAAGGTCGTATGCTTCGGTTAGCAGCAGCCTGTTTATCTGGTTCCAACTTCCGCTGAATCACAACCAGTTGTCCCTGAGCTATGACAAGAGAGGAAACAAAAAGTTAAAGGAGCAAGCCTGCCATACACCAGAAGCAAACTTCCTTGATAACATGCTTTTGCGAAGTTCAGGAAAATTAAATGTGGGCACCAAGAAAGAGGATGGTGAGAGTACAGCCCCCACCCCTCGTCCAAAGATCTTGCGATGTAAATGCCACCACCACTGTCCAGAA |
