# Supplementary material for: A unique 15-bp InDel in the first intron of BMPR1B regulates its expression in Taihu pigs
Source: BMC Genomics. 2022 Dec 3;23:799. doi: 10.1186/s12864-022-08988-6 (PMC9719134; doi:10.1186/s12864-022-08988-6)
Supplement: Supplementary file 4 — Additional file 4 : Table S4. The results of promoter predictions [file 12864_2022_8988_MOESM4_ESM.docx]

Table S4 The results of promoter predictions

| Promoter name | Strat code | Stop code | Score | Sequence |
| --- | --- | --- | --- | --- |
| Promoter 1 | 1939 | 1989 | 0.86 | ACCTGTTGGAAATATAAATTCTGATGTCCCAAGCCAGATCTACTGAATCA |
|  | 2226 | 2276 | 0.99 | GTTAGGAAAATATAAAAATGCCGCCACATCTAACAATGTGATTTAATCCA |
| Promoter 2 | 133 | 183 | 0.95 | CCTGAATTTTTAAAAAATCGGAAACAAAACTTAATAAATGTGATGGAGTT |
|  | 1005 | 1055 | 0.96 | AATGTAGGTGTAAATAATACCTGCTCCGTCGAGTCGTAATGGGAATTTAA |
|  | 1176 | 1226 | 0.99 | ATACTGTCCTTTAATAAGGCCCCTAAAACAGTCCCATTATCTAGCCATTA |
|  | 1189 | 1239 | 0.93 | ATAAGGCCCCTAAAACAGTCCCATTATCTAGCCATTATCCTTTGTCTACA |
|  | 1383 | 1433 | 0.99 | AAAAAAAAAAAAAAAAAGGGGGATTCTTAACCTTTCCCGATAGTCTCAGA |
|  | 1645 | 1695 | 0.89 | AGTTAGTGCTATAAACCCAGGCATCAGATAATCCTGATACATGAGATGAG |
|  | 1909 | 1959 | 0.99 | ACCTGGTGCTTTAAATATCCCCAGCTTAAGCTGCTTTCCCAGGCTGCTGT |
| Promoter 3 | 115 | 165 | 0.87 | CACAGAGCAGTTTATATGGGCCAGGCACTGTTCTAAGCACTTTATAAGTA |
|  | 272 | 322 | 0.90 | ATTGGGAAGTAATAAAGCTGGGGTCAAGACTGAGGAAATTGGAACAATAG |
| Promoter 4 | 575 | 625 | 0.93 | TAGACTCATCAATAAATGGAGCTCCACTGGCTCCTAAGTGGAAGTTGCAT |
|  | 1806 | 1856 | 1.00 | TGAGAGGGGAAAAAAAAAGTCCCGCGGCGGCGGCGGCGGCGGCAGAGCGG |
|  | 1971 | 2021 | 0.86 | TGCCGGGGCGCAGCGGCGGGTAAGGCGCGCGCGGGCGGGCGGCCGGCCGA |
