# Supplementary material for: A unique 15-bp InDel in the first intron of BMPR1B regulates its expression in Taihu pigs
Source: BMC Genomics. 2022 Dec 3;23:799. doi: 10.1186/s12864-022-08988-6 (PMC9719134; doi:10.1186/s12864-022-08988-6)
Supplement: Supplementary file 5 — Additional file 5 : Table S5. The information of primers used to construct plasmids in this study [file 12864_2022_8988_MOESM5_ESM.docx]

Table S5 The information of primers used to construct plasmids in this study

| Primer | Enzyme restriction sites | Sequences(5’-3’) | Anneal  (℃) | Usage |
| --- | --- | --- | --- | --- |
| P1 | NheI | F: CG*GCTAGC*GCTGAAGGCAAGAATGGGGAGT | 69 | Promoter activity |
| P1 | XhoI | R: TAA*CTCGAG*TGTGATGTAGGCTGGCAGCTAGAG | 69 | Promoter activity |
| P2 | NheI | F: CG*GCTAGC*CTGAAAGAGTAGAGGTGGATGT | 67 | Promoter activity |
| P2 | HindIII | R: CCC*AAGCTT*CCAAACCTTAATCTCCCAGTC | 67 | Promoter activity |
| P3 | NheI | F: CG*GCTAGC*TTACTAAAGGCACAGGGGCAGGA | 69 | Promoter activity |
| P3 | XhoI | R:TAA*CTCGAG*CGAGAGAAAGAGAGAGAGGGAGGG | 69 | Promoter activity |
| P4 | NheI | F: CG*GCTAGC*GACAAACCAAACACTCCCTGCTC | 69 | Promoter activity |
| P4 | HindIII | R: CCC*AAGCTT*TCAACTTCTGGCCGCCGTGG | 69 | Promoter activity |
| P1-ERE | NheI | F: CGGCTAGCAACCGACCTGCGTTTTCTTTTC | 68 | Expression regulation |
| P1-ERE | NheI | R: CGGCTAGCTCTTCCCTTCCTACCACCCTCCT | 68 | Expression regulation |
| P3-ERE | SacI | F: TAAGAGCTCAACCGACCTGCGTTTTCTTTTC | 67 | Expression regulation |
| P3-ERE | NheI | R: CGGCTAGCTCTTCCCTTCCTACCACCCTCCT | 67 | Expression regulation |
| P4-ERE | NheI | F: CGGCTAGCAACCGACCTGCGTTTTCTTTTC | 68 | Expression regulation |
| P4-ERE | Nhe | R: CGGCTAGCTCTTCCCTTCCTACCACCCTCCT | 68 | Expression regulation |
| ESR1 | - | F:TAAGCTAGCCGGGGGGAAACGGTTTGCGCCTTGC |  | Over expression |
| ESR1 | - | R: TGCTCTAGATCAGATTGTGGTGGGGAAGTTCT |  | Over expression |
| ESR1-cut | NheI | F: CGGCTAGCGGGCTGTGCTCTTCTTCCAGGTGGC |  | Over expression |
| ESR1-cut | BamHI | R: CGGGATCCTCAGATTGTGGTGGGGAAGTTCT |  | Over expression |
